# Serum lipid profile as an indicator of disease severity in chronic liver disease: A hospital-based cross-sectional study

**DOI:** 10.6026/973206300223131

**Published:** 2026-05-31

**Authors:** Vikas Rangare, Jyoti Nagwanshi, Kapil Raghuwanshi

**Affiliations:** 1 Department of Medicine, Chhindwara Institute of Medical Sciences, Chhindwara, Madhya Pradesh, India; 2 Department of Biochemistry, Chhindwara Institute of Medical Sciences, Chhindwara, Madhya Pradesh, India

**Keywords:** Chronic liver disease (CLD), lipid profile, low-density lipoprotein (LDL), high-density lipoprotein (HDL), cholesterol, child-pugh score

## Abstract

Chronic liver disease (CLD) is associated with significant metabolic alterations, particularly affecting lipid synthesis and regulation, making serum lipid levels potential indicators of disease severity. This cross-sectional, hospital-based study enrolled 120 adult CLD patients (≥18 years) of diverse etiologies, viral hepatitis B/C, alcoholic liver disease and non-alcoholic fatty liver disease (NAFLD)-to evaluate associations between serum lipid parameters and liver dysfunction. Fasting total cholesterol, LDL, HDL and triglycerides were measured and composite lipid scores were derived and analyzed against Child-Pugh classification using ANOVA, correlation and chi-square tests. Total cholesterol, LDL and HDL significantly declined with increasing Child-Pugh class (p < 0.001; η^2^ = 0.33-0.36), showing strong negative correlations with disease severity, whereas triglycerides decreased non-significantly (p = 0.072). The composite lipid score revealed robust discriminatory power (F = 68.2, p < 0.001, η^2^ = 0.54) irrespective of gender or etiology. Thus, we show serum lipid profiling particularly composite lipid scoring as a practical and cost-effective adjunct for assessing CLD severity and prognosis, especially in resource-limited settings.

## Background:

CLD remains significant health issue across world, with significant contribution to morbidity and mortality. It includes spectrum of progressive hepatic conditions, including chronic hepatitis, fibrosis, cirrhosis and subsequent liver failure. In most developing areas, patients often report at advanced stages of disease, which limits therapeutic interventions and results in worse outcomes [1]. The most asymptomatic nature of early CLD emphasizes importance of reliable ways to measure severity of disease and provide timely management. Liver plays crucial role in lipid metabolism as it is responsible for lipid synthesis, storage and transport. It controls synthesis of cholesterol, triglycerides, lipoproteins of high-density lipoprotein (HDL) and low-density lipoprotein (LDL). In case of hepatic dysfunction, processes are disrupted, which leads to changes in circulating lipid levels [2]. These alterations can be indicators of some underlying hepatic dysfunction as opposed to incidental observations. Previous research revealed that patients with CLD, especially those with cirrhosis, usually exhibit lower levels of total cholesterol, LDL, HDL. These findings are commonly attributed to decreased hepatic production, impaired lipoprotein metabolism, nutritional deficiency in advanced disease [3]. On other hand, triglyceride levels have shown variable patterns in research, with some reporting reduction and others showing minimal or variable effect [4]. Such variations can be impacted by differences in patient population, underlying etiologies, related metabolic conditions. Assessment of disease severity is important part of CLD management and it has direct impacts on prognosis and decision-making in therapeutic treatment. Child-Pugh is one of most common clinical tools that have increased clinical and biochemical parameters to classify patients into various levels of severity [5]. Nevertheless, certain aspects of this scoring system are subjective, which promotes additional objectives to find more markers which will help to improve disease evaluation. In this context, serum lipid profiling could be effective but underutilized methodology.

Generally, lipid parameters are often cost-effective, accessible, frequently measured in resource-constrained areas. Since liver functions and lipid metabolism are closely related physiologically, these parameters could provide additional insights into severity of disease [6]. Previous studies have investigated relationship between lipid level and liver disease severity and some studies have indicated that cholesterol and hepatic impairment have negative correlation. It has been reported that lower lipid levels were linked to more advanced disease and poor clinical outcomes [7]. But, inconsistencies in results as well as differences among studies in terms of design and patient characteristics, limit definitive conclusions, suggesting further research. This study will examine whether lipid profiles can be simple and practical predictor of disease progression in CLD by focusing on biochemical parameters which are routinely available [8]. Therefore, it is of interest to determine serum lipid levels in patients with CLD and to investigate how they are related to severity of disease based on Child-Pugh classification.

## Materials and Methods:

## Study design and setting:

This hospital-based cross-sectional research has been conducted after obtaining Institutional Human Ethics Committee approval (approval number: CIMS/Ethics Committee/2024/8515, dated 01/08/2024). This has been performed in Department of Medicine and Biochemistry at Chhindwara Institute of Medical Sciences associated with District Hospital Chhindwara in Madhya Pradesh, India, for one year (December 2024 to November 2025).

## Study population:

Random enrollment of adult patients (≥18 years) was used to minimize risk of selection bias and obtain representativeness in terms of disease severity. Eligibility was received after clinical examination, biochemical analysis, imaging results. Viral hepatitis B/C, alcoholic liver disease (ALD), non-alcoholic fatty liver disease (NAFLD) was also taken as common etiologies of CLD to provide broad clinical presentation of condition.

## Sample size:

Based on earlier study that showed moderate correlations (r ≈ 0.3) between serum lipid parameters and Child-Pugh scores [9, 10], minimum sample size of 85 was estimated that would achieve 80% power at 5% significant level. The sample size was raised to 120 to obtain adequate representation in Child-Pugh categories and to examine subgroups.

## Inclusion and exclusion criteria:

## Inclusion criteria:

Adult's ≥18 years with confirmed CLD of any of the above etiologies.

## Exclusion criteria:

Acute liver failure, significant renal impairment, poorly controlled diabetes mellitus, active systemic infections, recent lipid-lowering treatment (within 3 months). These criteria were used to reduce confounding effects on lipid metabolism or severity of disease assessment (e.g., metabolic status, use of medication).

## Data collection and clinical assessment:

Baseline demographics (age, sex), disease etiology, duration of illness, comorbidities were noted. A systematic clinical examination was done to determine signs of chronic liver dysfunction, such as icterus, ascites, hepatosplenomegaly, hepatic encephalopathy. Child-Pugh scoring system was used to classify severity of diseases as it is based on parameters in laboratory (bilirubin, albumin and prothrombin time), clinical aspects (ascites, encephalopathy). Clinical assessment was consistent and reliable through standardized procedures [11].

## Biochemical analysis:

Fasting venous blood samples were measured (8-12 hours), total cholesterol, LDL, HDL, triglycerides were performed in standardized enzymatic spectrophotometric assays on automated analyzer (Biosystem BA 400). A combined lipid score was determined by following formula to measure total lipid alteration:

Combined lipid score = Z Total cholesterol + Z LDL + Z HDL

Where each -score demonstrates standardized value of respective lipid parameter [12].

The composite score is measure of overall lipid derangement and hence single-composite measure of lipid changes in disease severity.

## Statistical analysis:

Continuous variables have been exhibited as mean ± SD and categorical variables have been displayed as counts and percentages. One-way ANOVA with Tukey post-hoc tests was utilized to compare Child-Pugh classes and etiologies. Independent samples t-tests have been utilized for comparing genders. Pearson's correlation coefficient determined association between lipid parameters, combined lipid score, Child-Pugh score. Chi-square test of trend was used to examine categorical lipid abnormalities by disease severity. Effect sizes were computed: η^2^ for ANOVA, Cohen's d for t-tests, Cramér's V for χ^2^ analyses. Statistical significance was declared as p < 0.05. Normality assumptions (Shapiro-Wilk test) and homogeneity of variances (Levene's test) have been observed to determine validity of parametric tests.

## Data quality assurance:

All laboratory analyses were carried out in certified clinical biochemistry laboratory and regular calibration of instruments and internal quality control were carried out using reference materials. Duplicate samples were analyzed. Two investigators entered data and cross-validated it against source records and determined that discrepancies were due to errors.

## Results:

The study involved total 120 adult patients with CLD. Mean age was 48.6 ± 12.4 years. Males comprised 65.0% (78/120; 95% confidence interval [CI]: 56.3%-73.1%), while females accounted for 35.0% (42/120; 95% CI: 26.9%-43.7%). Patients were distributed across Child-Pugh classes A (34, 28.3%; 95% CI: 20.5%-37.3%), B (46, 38.3%; 95% CI: 29.6%-47.7%) and C (40, 33.4%; 95% CI: 25.3%-42.4%). In terms of etiology, viral hepatitis B/C was most frequent (54, 45%), followed by ALD (36, 30%) and NAFLD (30, 25%) (Table 1). Cohort demonstrates wide clinical spectrum, but its hospital-based nature can be limitation to generalization. Serum lipid parameters indicated progressive decline with increasing severity of disease (Table 2). Total cholesterol (F (2,117) = 32.5, p < 0.001, η^2^ = 0.36), LDL (F (2,117) = 28.7, p < 0.001, η^2^ = 0.33) and HDL (F (2,117) = 30.1, p < 0.001, η^2^ = 0.34) demonstrated statistically significant differences, with substantial effect sizes. Post hoc Tukey was used to ensure that all pairwise comparisons were statistically significant. Triglycerides showed a decreasing trend; however, this did not reach statistical significance (F (2,117) = 2.7, p = 0.072, η^2^ = 0.04), exhibiting small effect. Pearson correlation analysis displayed inverse correlation between lipid parameters and disease severity (Table 3). Total cholesterol showed a moderate-to-strong negative correlation with Child-Pugh score (r=-0.62; 95% CI: -0.72 to -0.49; p<0.001). Similarly, LDL (r=-0.58; 95% CI: -0.69 to -0.44; p<0.001) and HDL (r=-0.55; 95% CI: -0.67 to -0.41; p<0.001) exhibited moderate negative correlations. Conversely, triglycerides observed weak negative correlation, though it has not been statistically significant (r = -0.18; 95% CI: -0.35 to 0.01; p=0.072). These results show that decline in cholesterol fractions has stronger correlation with progressive severity of liver dysfunction as compared to triglycerides. Lipid parameters were compared in males and females and found to show no statistically significant differences (Table 4). Effect sizes have been negligible (Cohen's d < 0.2 for all parameters), which showed no statistically significant difference between genders. There have been no statistically significant differences in lipid parameters among etiological groups (viral hepatitis, ALD, NAFLD) (Table 5). Effects were not significant (η^2^ ≤ 0.03) and indicated limited influence of etiology on lipid levels in this cohort. Combined lipid score demonstrated progressive reduction as disease severity advanced (Table 7). This difference was significantly different (F (2, 117) = 68.2, p<0.001) with very high effect size (η^2^=0.54), which demonstrated considerable differentiation among Child-Pugh classes. Lipid abnormalities were significantly increased as prevalence of Child-Pugh class worsened (Table 6). Total cholesterol <150 mg/dL, low LDL, low HDL became increasingly common in advanced disease with moderate-to-large effect sizes (Cramer's V varying from 0.35-0.44). Together, increase in severity of CLD significantly reduced level of Serum total cholesterol, LDL, HDL showing significant effect sizes and strong inverse correlations. Associations between triglycerides were weak and non-significant. There were no clinically meaningful differences by gender or disease etiology. The combined lipid score showed clear difference among stages of disease. These results indicate that lipid parameters, specifically cholesterol fractions, are connected with severity of disease, but these interpretations must be considered in context of cross-sectional design, sampling in hospital, potential confounding factors.

## Discussion:

In this study of 120 adult patients with CLD, we observed a consistent inverse relationship between serum lipid parameters and disease severity. Total cholesterol, LDL, HDL levels declined progressively from Child-Pugh class A to C, with large effect sizes (η^2^ = 0.33-0.36), while triglycerides showed only a mild, non-significant decline (η^2^ = 0.04). Pearson correlation revealed moderate-to-strong negative correlations between Child-Pugh scores and total cholesterol (r=-0.62), LDL (r=-0.58), HDL (r=-0.55), but triglycerides did not show significant negative correlation (r = -0.18). These results support idea that cholesterol fractions, rather than triglycerides are reliable measures of hepatic dysfunction and disease progression [13]. Liver plays critical role in lipid homeostasis involving production of cholesterol, apolipoproteins lipoprotein particles and clearance of lipids in circulation. Hepatocellular injury interferes with these mechanisms causing decrease in circulating cholesterol levels and alterations in lipoprotein composition. Mechanistic research shows that reduced activities of enzymes like lecithin-cholesterol acyltransferase and reduced apolipoprotein production are contributing factors to hypocholesterolemia in cirrhosis. Besides, advanced CLD damages lipid metabolism through systemic inflammation and oxidative stress, which additionally influences lipoprotein composition and clearance [14, 15, 15]. Mechanisms are supported by clinical studies. Yamuna *et al.* showed progressive reductions in total cholesterol, LDL, HDL with increasing Child-Pugh and MELD scores that confirmed association between lipid derangement and hepatic dysfunction [13]. Similar results were obtained by Sharma *et al.* who identified that low lipid fractions correlated with higher disease severity, which may highlight potential of lipid profiles as adjunct markers of CLD [14]. Badawi *et al.* showed that changes in cholesterol and HDL were reliably related to hepatic dysfunctions in various etiologies [15]. Deprince *et al.* highlighted mechanistic connections between dysregulated lipid metabolism and liver disease progression, which depict systemic effects of hepatic impairment [16]. These patterns are supported by etiology-specific analyses. Our cohort found no significant differences between etiologies (viral hepatitis, ALD, or NAFLD; η^2^ ≤ 0.03) and overall tendency of decreasing cholesterol fractions with severity of disease remained. It means that force behind lipid change is not underlying disease origin but hepatic dysfunction. These outcomes are similar to those of other reports by Doshi *et al.* and Mujahid *et al.* who found lower total cholesterol, HDL, LDL in advanced cirrhosis across various etiologies [17, 18]. Triglycerides were less consistent, with mild non-significant decrease, which aligns with previous observations [19]. Manjesh *et al.* [20] also found that lipid concentrations decline as cirrhosis severity increases, with total cholesterol, LDL, VLDL, triglycerides and HDL markedly lower in patients with advanced Child-Pugh class and complications such as ascites or spontaneous bacterial peritonitis. These lipid abnormalities likely reflect impaired hepatic lipoprotein synthesis and metabolism and could serve as complementary prognostic markers alongside Child-Pugh and MELD scores, meriting prospective validation [21]. Dietary intake, insulin resistance, systemic inflammation may lead to variability in triglyceride levels and limit their application as indicator of severity of liver disease. Conversely, combined lipid score, which was calculated from standardized z-scores of total cholesterol, LDL, HDL exhibited significant decrease with advancement of disease (F (2,117) = 68.2, η^2^ = 0.54), proving that integrated lipid metric robustly indicates hepatic dysfunction and disease severity. Emerging lipidomic studies also support this prognostic ability of serum lipids. Hornemann (2022) determined certain lipid species that might become sensitive biomarkers of hepatocellular injury and fibrosis and might be more accurate than conventional lipid panels [22]. Arain *et al.* showed that integrating lipid biomarkers with traditional clinical parameters could be used to improve prognostic accuracy in CLD [23]. Tyagi *et al.* [24] also found that patients with chronic (predominantly alcoholic) liver disease had significantly lower total cholesterol, LDL, HDL and triglycerides than healthy controls(p < 0.05), with greater lipid derangement in higher Child-Pugh classes. These findings support using routine lipid profiling in cirrhotic patients as a simple adjunctive marker of hepatic dysfunction and to guide clinical management [24]. These results demonstrate potential of serum lipid determinations as accessible, low-cost adjuncts to conventional risk assessment and as candidates for advanced biomarker identification. Strengths of study include well-characterized hospital cohort balances represented by Child-Pugh classes and etiologies, use of standardized biochemical approach and comprehensive statistical analyses that incorporate assessment with regard to integrated lipid score. The study is cross-sectional and single-centered, eliminating causal inference and generalization. Diet, insulin resistance, systemic inflammation, additional possible confounders, including metabolic condition and use of medication were not completely controlled. These factors have effect on serum triglycerides. Large effect size, multiple tests, strong statistical significance is positive indicators of validity of findings. The findings indicate that serum lipid parameters especially total cholesterol, LDL; HDL levels could be cost-effective adjunctive markers of disease severity in CLD. They may be utilised to highlight risks at earlier stages of treatment, guide therapeutic decisions, aid in management of disease progression, particularly in resource-constrained environments. Further research should also focus on priority of multicenter, longitudinal studies to confirm prognostic value of lipid profiles and explain how they can be applied to complex frameworks of risk assessment. Moreover, cutting-edge lipidomic studies have potential to identify new biomarkers to diagnose earlier and monitor disease development, contribute to individual patient-based approaches to management.

## Conclusion:

This hospital-based study of 120 adults with chronic liver disease showed that serum total cholesterol, LDL and HDL levels significantly declined with increasing Child-Pugh class, showing moderate-to-strong inverse correlations, while triglycerides exhibited a mild, non-significant decrease. Lipid alterations were consistent across etiologies and genders, suggesting hepatic dysfunction as the primary determinant rather than disease origin. The combined lipid score proved highly discriminatory for disease severity, confirming traditional lipid profiling as a practical adjunct for evaluating hepatic impairment and prognosis, with attention to confounding factors such as metabolic state and medication use.

## Figures and Tables

**Table 1:** Baseline characteristics of the study population

**Variable**	**Total (n = 120)**	**95% CI**
Age (years)	48.6 ± 12.4	-
Male, n (%)	78 (65.0)	56.3-73.1
Female, n (%)	42 (35.0)	26.9-43.7
Child-Pugh Class A	34 (28.3)	20.5-37.3
Child-Pugh Class B	46 (38.3)	29.6-47.7
Child-Pugh Class C	40 (33.4)	25.3-42.4
Viral Hepatitis B/C	54 (45.0)	-
Alcoholic Liver Disease	36 (30.0)	-
Non-Alcoholic Fatty Liver Disease	30 (25.0)	-

**Table 2:** Serum lipid profile across child-pugh classes

**Parameter**	**Class A**	**Class B**	**Class C**	**F (2,117)**	**p-value**	**η^2^**	**Mean Difference (A vs C)**	**95% CI**
Total Cholesterol	162.5 ± 28.7	138.2 ± 25.4	112.6 ± 21.8	32.5	<0.001	0.36	49.9	38.1-61.7
LDL	98.4 ± 22.1	82.7 ± 19.6	65.3 ± 17.2	28.7	<0.001	0.33	33.1	23.7-42.5
HDL	42.3 ± 6.8	36.5 ± 5.9	30.8 ± 5.4	30.1	<0.001	0.34	11.5	8.6-14.4
Triglycerides	142.5 ± 35.6	135.7 ± 33.2	128.9 ± 30.1	2.7	0.072	0.04	13.6	-2.1-29.3

**Table 3:** Correlation of lipid parameters with child-pugh score

**Parameter**	**r**	**95% CI**	**p-value**
Total Cholesterol	-0.62	-0.72 to -0.49	<0.001
LDL	-0.58	-0.69 to -0.44	<0.001
HDL	-0.55	-0.67 to -0.41	<0.001
Triglycerides	-0.18	-0.35 to 0.01	0.072

**Table 4:** Lipid parameters according to gender

**Parameter**	**Male (Mean± SD)**	**Female (Mean± SD)**	**Mean Difference**	**95% CI**	**p-value**	**Cohen's d**
Total Cholesterol	135.8± 32.1	139.5± 29.4	-3.7	-13.4 to 6.0	0.45	0.12
LDL	81.4± 20.8	84.2± 19.2	-2.8	-10.6 to 5.0	0.38	0.14
HDL	36.1± 6.7	37.2± 5.8	-1.1	-3.2 to 1.0	0.31	0.17
Triglycerides	136.5± 32.5	133.8± 31.2	2.7	-8.9 to 14.3	0.54	0.08

**Table 5:** Lipid parameters according to etiology of chronic liver disease

**Parameter**	**F(2,117)**	**p-value**	**η^2^**
Total Cholesterol	1.75	0.18	0.03
LDL	1.62	0.2	0.03
HDL	1.48	0.23	0.02
Triglycerides	1.69	0.19	0.03

**Table 6:** Prevalence of lipid abnormalities by child-pugh class

**Parameter**	**χ^2^(df=2)**	**p-value**	**Cramér's V**
Total Cholesterol <150 mg/dL	45.6	<0.001	0.44
LDL <70 mg/dL	32.8	<0.001	0.37
HDL <35 mg/dL	29.4	<0.001	0.35

**Table 7:** Combined lipid score according to disease severity

**Child-Pugh Class**	**Mean ± SD**
Class A	1.2 ± 0.5
Class B	-0.3 ± 0.6
Class C	-1.5 ± 0.7
ANOVA: F (2,117) = 68.2,
p < 0.001, η^2^ = 0.54.
Mean Difference (A vs C):
2.7 (95% CI: 2.3-3.1)
